# Mechanosensitive TRPM7 mediates shear stress and modulates osteogenic differentiation of mesenchymal stromal cells through Osterix pathway

**DOI:** 10.1038/srep16522

**Published:** 2015-11-12

**Authors:** Yi-Shiuan Liu, Yu-An Liu, Chin-Jing Huang, Meng-Hua Yen, Chien-Tzu Tseng, Shu Chien, Oscar K. Lee

**Affiliations:** 1 Stem Cell Research Center, National Yang-Ming University, Taipei 11221, Taiwan; 2 Institute of Engineering in Medicine, University of California at San Diego, La Jolla, CA 92093, USA; 3 Departments of Bioengineering and Medicine, University of California at San Diego, La Jolla, CA 92093, USA; 4 Taipei City Hospital, Taipei 10341, Taiwan; 5 Institute of Clinical Medicine, National Yang-Ming University, Taipei 11221, Taiwan; 6 Department of Medical Research, Taipei Veterans General Hospital, Taipei 11217, Taiwan

## Abstract

Microenvironments that modulate fate commitments of mesenchymal stromal cells (MSCs) are composed of chemical and physical cues, but the latter ones are much less investigated. Here we demonstrate that intermittent fluid shear stress (IFSS), a potent and physiologically relevant mechanical stimulus, regulates osteogenic differentiation of MSCs through Transient receptor potential melastatin 7 (TRPM7)-Osterix axis. Immunostaining showed the localization of TRPM7 near or at cell membrane upon IFSS, and calcium imaging analysis demonstrated the transient increase of cytosolic free calcium. Expressions of osteogenic marker genes including Osterix, but not Runx2, were upregulated after three-hour IFSS. Phosphorylation of p38 and Smad1/5 was promoted by IFSS as well. TRPM7 gene knockdown abolished the promotion of bone-related gene expressions and phosphorylation. We illustrate that TRPM7 is mechanosensitive to shear force of 1.2 Pa, which is much lower than 98 Pa pressure loading reported recently, and mediates distinct mechanotransduction pathways. Additionally, our results suggest the differential roles of TRPM7 in endochondral and intramembranous ossification. Together, this study elucidates the mechanotransduction in MSCs fate commitments and displays an efficient mechano-modulation for MSCs osteogenic differentiation. Such findings should be taken into consideration when designing relevant scaffolds and microfluidic devices for osteogenic induction in the future.

Physiologically, shear stress is generated by blood flow and interstitial fluid. When bones are mechanically loaded, a thin layer of interstitial fluid flow is generated due to pressure gradients caused by deformation of mineralized matrices and porous structures such as bone or medullary cavity. Shear stress, with the magnitude varying from 8 to 30 dyne/cm^2^ (0.8 to 3 Pa), exerted by different physical activities^1^, crosses bone cells as well as precursor cells and plays a role in bone homeostasis^2^,^3^. Mesenchymal stromal cells (MSCs), rare but important cell population residing in the bone marrow and other somatic tissues, are regulated by shear stress during the processes of osteogenic, chondrogenic, and endothelial differentiations^4^,^5^,^6^. Moreover, because of their potential to differentiate into different cell types, MSCs are widely used in cell therapy and tissue engineering^7^,^8^. It is therefore important to understand how MSCs sense shear stress and what are the underlying molecular mechanisms regulating MSC fate commitments, particularly in tissue regeneration.

Transient receptor potential cation channel (TRP channel), subfamily M, member 7 (TRPM7) is one of the only two known TRP channels possessing a regulatory kinase domain at its carboxyl terminus^9^,^10^,^11^. TRPM7 regulates calcium and magnesium homeostasis^12^, actomyosin contractility, and cell adhesion^13^. *In vivo* studies also showed that TRPM7 is important in embryonic development^14^ and bone formation. For example, Zebrafish trmp7-mutant displayed defects in skeletogenesis, including accelerated endochondral ossification and delayed intramembranous ossification^15^. It has been reported that TRPM7 mediated the shear flow-induced calcium current in fibroblasts and osteoblasts^16^,^17^. Recently, using patch clamp techniques, Xiao *et al.* also demonstrated that TRPM7 is mechanosensitive to pressure loading of 1 g/cm^2^ (98 Pa), induces cytosolic Ca^2+^ increase and upregulates Runx2 gene in human MSCs^18^. However, the underlying mechanotransduction pathways for osteogenic differentiation triggered by TRPM7 activation in other physiological condition are still illusive. In the present study, by applying intermittent fluid shear stress (IFSS) ranging from 4 × 10^−3^ to 1.2 Pa in detachable microfluidic devices, we demonstrated that mechanosensitive TRPM7 modulates osteogenic differentiation of MSCs through Osterix pathway via kinase phosphorylation, intracellular calcium increase, and upregulation of the osteogenic marker genes upon the mechanical cue. The study has shed light on the involvements of mechanosensors in MSC fate commitments and displays an efficient mechano-modulation of MSCs osteogenic differentiation.

## Results

### Short duration of intermittent shear flow modulated osteogenic differentiation of MSCs

**Figure 1:**
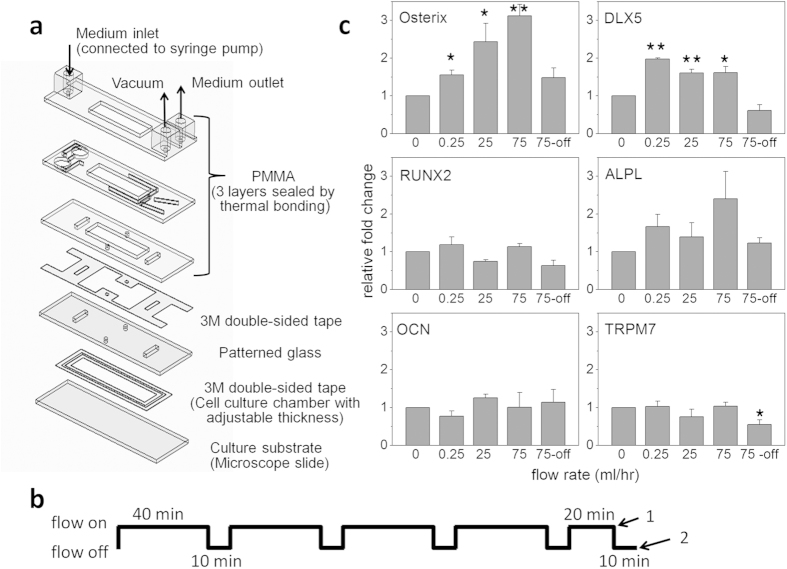
Shear stress regulated osteogenic differentiation of MSCs. (**a**) Components of the microfluidic devices for applying fluid shear stress. (**b**) Intermittent fluid flow pattern was used for the study. Cells were harvested when flow was on at time point 1 (arrow 1) and when flow was off at time point 2 (arrow 2). (**c**) Relative expressions of osteogenic marker genes of MSCs under 0 ml/hr, 0.25 ml/hr, 25 ml/hr, and 75 ml/hr fluid flow for three hours with total of intermittent shear stress (IFSS) on-off cycle lasting for 230 minutes (time point 1), as well as under 75 ml/hr fluid flow but of the cells harvested when flow was off with total of IFSS cycle lasting for 240 minutes (75 ml/hr-off at time point 2). The flow rates were equal to 0, 0.04, 4, and 12 dyne/cm^2^, or 0, 0.004, 0.4, and 1.2 Pa of shear stress respectively. Gene expressions were analyzed by RT-PCR. Data were normalized by the respective gene expressions of MSCs under flow rate 0 ml/hr (static control) and presented as fold change ± SEM (n = 4). Significant difference *indicated p < 0.05 and **indicated p < 0.005 from values obtained in the cells of static control. ALPL: Alkaline phosphatase. COL1A1: Collagen type I alpha 1. Dlx5: Distal-less homeobox 5. OCN: Osteocalcin. Runx2: Runt-related transcription factor 2. (Figure 1a drawn by Meng-Hua Yen).

It has been reported that pause insertion during consecutive mechanical loading can induce substantial bone formation *in vivo*^19^,^20^. *In vitro* study also demonstrated that intermittent fluid shear flow (IFSS) induces the osteogenic differentiation of MSCs more efficiently compared to continuous one after four-day stimulation. The advantage of the intermittent over continuous mechanical stimulation may be attributable to the adaptive response of cells under continuous mechanical stress^21^. Therefore, we employed an intermittent, unidirectional flow pattern to amplify the early responding signals of MSCs to the mechanical stimulation. The on/off cycle was 40 min/10 min and the total duration of “stress-on” was three hours (Fig. 1b). Flow rates used were 0.25 ml/hr, 25 ml/hr, and 75 ml/hr, which exerted on wall with shear stresses of 0.004 Pa, 0.4 Pa, and 1.2 Pa respectively. The shear stress exerted on cells is expected to be slightly higher than the wall shear stress, but the values were still within the physiological range since the size of MSCs was relatively small compared to that of flow chamber^22^. RT-PCR results on cells harvested during flow showed that gene expression of osteogenic transcription factor Osterix increased in a flow-rate dependent manner and the upregulation was responsive to the intermittent shear stress as low as 4 × 10^−3^ Pa. Upregulation of Osterix was diminished when the cells were harvested 10 min after shear flow was turned off (Fig. 1c and Table 1). Gene expression of distal-less homeobox 5 (Dlx5), another transcription factor needed for osteoblast differentiation, was sensitive to flow-on and flow-off as well. In contrast, gene expression of Runt-related transcription factor 2 (Runx2) did not significantly change after three hours of IFSS, unlike its upregulation during continuous flow (Supplementary Figure S1).

Osterix is a zinc finger-containing transcription factor that is essential for osteogenic differentiation and bone formation. It induces the expressions of collagen type I alpha-1 (COL1A1) and alkaline phosphatase (ALPL). Runx2 is also an osteogenic marker gene and has the bipotential in regulating both osteoblast and chondrocyte differentiation^23^,^24^. Additionally, morphology and Alkaline Phosphatase staining of MSCs after three-day induction demonstrate that MSCs pre-treated with three hours of IFSS exhibited more osteoblast-like phenotypes compared to the ones without IFSS (Supplementary Figure S2). The results indicate that a short duration of IFSS was able to promote the osteogenic fate commitment of MSCs through modulating Osterix expression and that the modulation was independent of Runx2.

### Short duration of intermittent shear flow increased TRPM7 protein expression and induced TRPM7 localization

**Figure 2:**
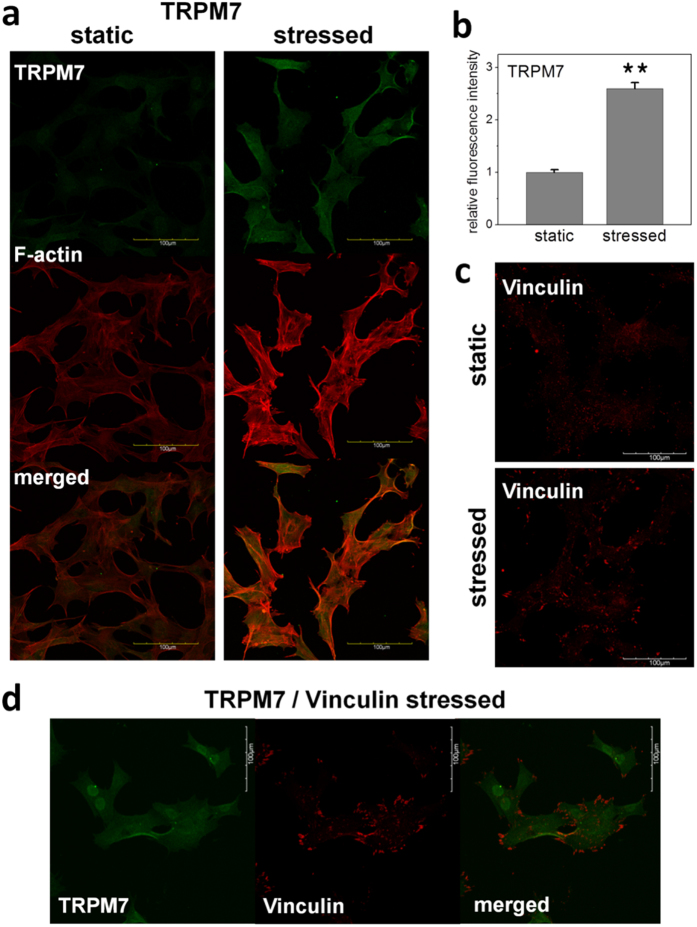
Shear stress induced localization of TRPM7 and vinculin. (**a**) TRPM7 protein expression represented by immunofluorescent staining in static and under shear stress condition was shown in green. F-actin was shown in red (Phalloidin). Stressed cells were under 75 ml/hr intermittent fluid flow for three hours. TRPM7 protein expression increased after 3 hours of intermittent FSS. Some of the TRPM7 were localized at the proximity of cell membrane. Scale bar = 100 μm. (**b**) Relative mean fluorescence intensities of TRPM7 of the cells in static control and under shear stress (N = 34). Data were normalized by the static control and presented as mean ± SEM. Significant difference **indicated p < 0.005. (**c**) Expression and localization of vinculin in static and under shear stress condition was shown in red. Shear flow enhanced the expression and spotty-localization of vinculin. (**c**) Immunofluorescent staining of TRPM7, vinculin, and the overlay of TRPM7 (green) and vinculin (red) under three hours intermittent shear stress.

We analyzed gene expressions of potential stretch-activated ion channels (SACs) in MSCs, including transient receptor potential ion channels TRPC1, TRPC6, TRPV1, TRPV4, and TRPM7, two-pore domain potassium channels TREKs and TRAAK, as well as acid-sensing ion channels ASICs. TRPM7 was one of the abundant ones among these cation channels in undifferentiated MSCs. In addition, TRPM7 was upregulated along the maturation process in the early stage of osteogenic differentiation of MSCs (Supplementary Figure S3). However, we did not observe a significant change in TRPM7 gene expression after 3 hours of IFSS. A previous study has reported the correlation between translocation of TRPM7 and shear flow in transfected overexpression system^25^. Therefore, we further investigated whether the endogenous TRPM7 protein in MSCs has a similar response to shear flow despite no upregulation in gene expression. Indeed, immunofluorescent staining showed that TRPM7 protein expression increased after a short duration of IFSS. Some of the TRPM7 were localized at the proximity of cell membrane, but there was no correlation between TRPM7 localization and flow direction (Fig. 2a). Meanwhile, shear flow also enhanced the expression of F-actin (Fig. 2a) and spotty-localization of vinculin, a cytoskeletal and focal adhesion-associated protein (Fig. 2c). The localization of TRPM7 and vinculin under shear flow shared some degree of overlap at the proximity of cell membrane (Fig. 2d). Our results demonstrated that TRPM7 was mechanosensitive to shear flow.

### TRPM7 knockdown and 2-APB blocking attenuated the upregulation of osteogenic marker genes after shear stress

**Figure 3:**
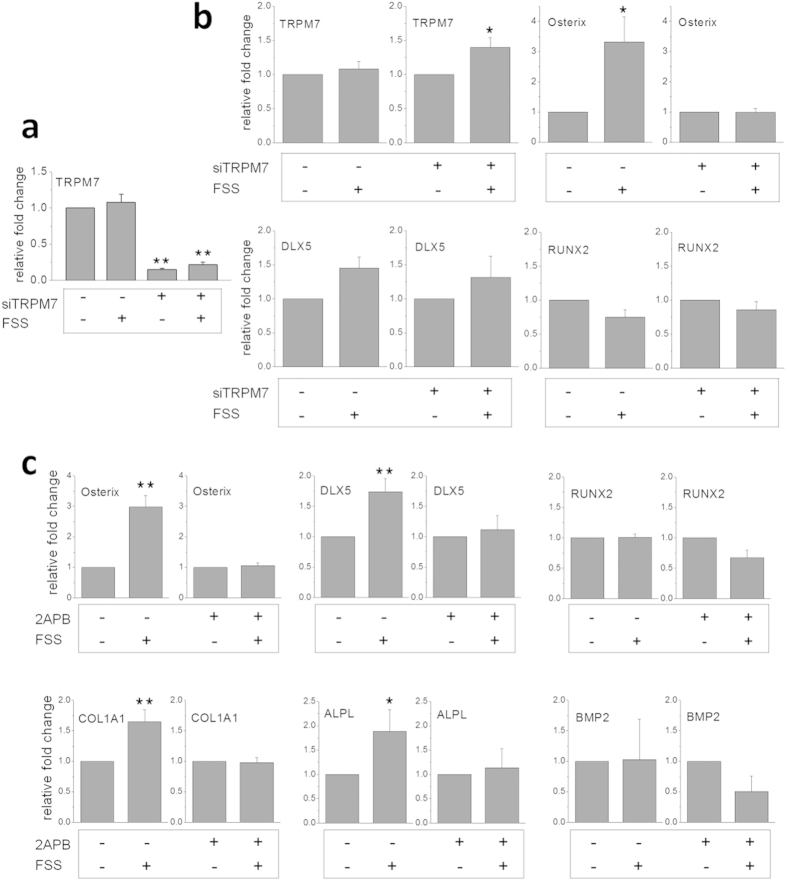
TRPM7 knockdown and blocking abolished the upregulation of osteogenic marker genes under shear flow. (**a**) Gene expressions of TRPM7 treated with scrambled siRNA (sham control) and TRPM7 siRNA were analyzed by RT-PCR. The expression of TRPM7 decreased to 20% compared to the sham control after siRNA treatment for 24 hours. (**b**) Relative gene expressions of TRPM7, Osterix, Runx2, and Dlx5 with and without intermittent shear stress. TRPM7 knockdown attenuated the upregulation of Osterix upon shear flow but with little effect on the gene expressions of Dlx5 and Runx2 compared to the sham control. (**c**) Relative gene expressions of Osterix, Dlx5, Runx2, COL1A1, ALPL, and bone morphogenetic proteins (BMP2) with and without 100 μM 2-APB additive were assayed by RT-PCR. 2-APB was able to diminish the upregulation of osteogenesis-related genes induced by three-hour IFSS. Data were normalized by the respective gene expressions of MSCs without FSS (static control) and presented as fold change ± SEM (n = 3). Significant difference *indicated p < 0.05 and **indicated p < 0.005. FSS: intermittent fluid shear stress.

Since TRPM7 expression and localization in the proximity of cell membrane was induced by 3 hours of IFSS and the upregulation of Osterix was mechanosensitive to flow on and off, we hypothesized that the localization initiated the mechanotransduction and resulted in the changes of osteogenic-specific gene expression. Therefore, TRPM7 was knocked down by small interfering RNA (siRNA), and the modulation of osteogenic-specific genes after the knockdown was assessed. RT-PCR results displayed that TRPM7 knockdown eliminated the upregulation of Osterix induced by IFSS and that the gene expression remained at the same level as if there were no shear flow. The expression patterns of transcription factors Runx2 and Dlx5 after knockdown were similar to those of the respective scrambled controls (Fig. 3a,b, and Supplementary Figure S4).

TRPM7 is essential for MSCs proliferation and maybe important to osteogenic differentiation^26^. In order to exclude the possibility that the diminish of Osterix upregulation was due to the stage of cell cycle MSCs were in as a result of TRPM7 gene knockdown, rather than the loss of mechanosensitivity, we performed the pharmacological blocking tests as well. 2-Aminoethoxydiphenyl borate (2-APB) is an activator of TRPV1, TRPV2, TRPV3, and TRPM6^27^ and a blocker of TRPC1, TRPC3, and TRPC6 channels at micromolar concentration^28^. 2-APB has been widely used as an inhibitor for investigating the function of TRPM7 as well. Since the transcript levels of above mentioned TRPCs genes are about hundred-fold less than that of TRPM7 in mouse MSCs, we used 2-APB to perform the pharmacological blocking test of TRPM7. Our results showed that 2-APB was able to diminish the upregulation of all osteogenesis-related genes that we have tested under IFSS, including Osterix, Dlx5, ALPL, and COL1A1 (Fig. 3c and Supplementary Figure S4). Thus, the results exhibited the important roles of TRPM7 in modulating osteogenic differentiation of MSCs in response to mechanical cues.

### Promotion of kinase phosphorylation by intermittent shear flow was mediated by TRPM7

**Figure 4:**
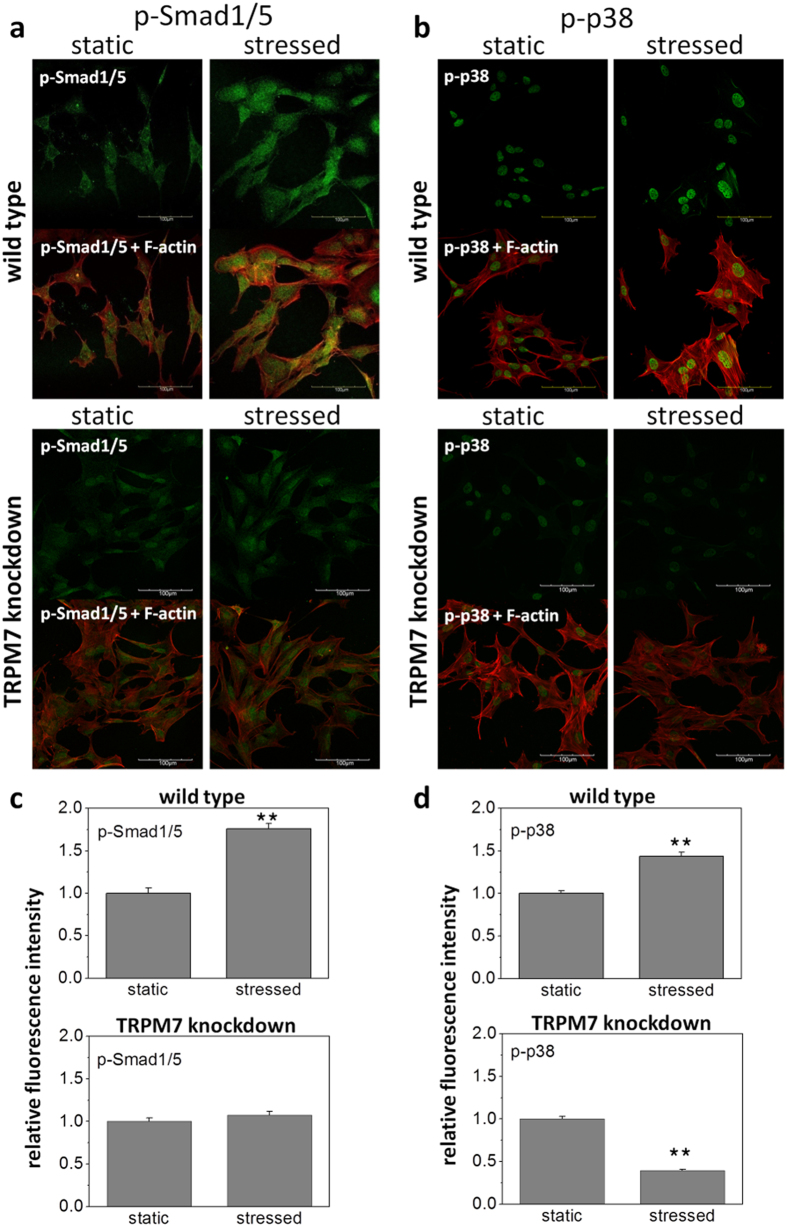
Shear stress induced phosphorylation of Smad1/5 and p38 MAPK. (**a, b**) Immunofluorescent staining of phosphorylated Smad1/5 (p-Smad1/5) and phosphorylated p38 MAPK (p-p38) was shown in green. F-actin was shown in red. Stressed cells were under 75 ml/hr intermittent fluid flow for three hours. Scale bar = 100 μm. (**c, d**) Mean fluorescence intensities of p-Smad1/5 (N = 27) and p-p38 (N = 68 for wild type and N = 42 for TRPM7 knockdown) of the cells in static culture and under shear stress. Data were normalized by the static control and presented as mean ± SEM. Significant difference *indicated p < 0.05 and **indicated p < 0.005 from the values obtained in the cells of static control.

TRPM7 is one of the only two TRP channels which possess alpha-kinase domain at their C-termini. We speculated that kinase activities of the channel can be activated by shear flow once the channel trans-located to cell membrane and subsequently triggered the downstream phosphorylation cascades. p38 MAPK has been shown to be associated with TRPM7-dependent ROS responses in fibroblasts^29^ and phosphorylated Smad1/5 has been shown to be upregulated by shear flow in osteosarcoma cell lines^30^. Moreover, both p38 MAPK and Smad1/5 are regulators of osteogenesis by phosphorylating Osterix^31^,^32^. Therefore, we tested whether IFSS could trigger the kinase activities of these two signaling pathways. After three-hour IFSS, both phosphorylated p38 and phosphorylated Smad1/5 exhibited a stronger nuclear immunofluorescent staining with 1.4-fold and 1.8-fold increases in mean fluorescence intensity, respectively (Fig. 4 and Supplementary Figure S5). The promotion of phosphorylation was diminished after TRPM7 knockdown. Similarly, the attenuation of IFSS promotion after TRPM7 knockdown was also observed by the enzyme-linked immnuosorbent assay (ELISA) (Supplementary Figure S5). Our results indicated that the kinase activity of TRPM7 was correlated with shear flow and important to the phosphorylation of Smad1/5 and p38 MAPK.

### Shear flow immediately induced transient increase of intracellular free calcium

**Figure 5:**
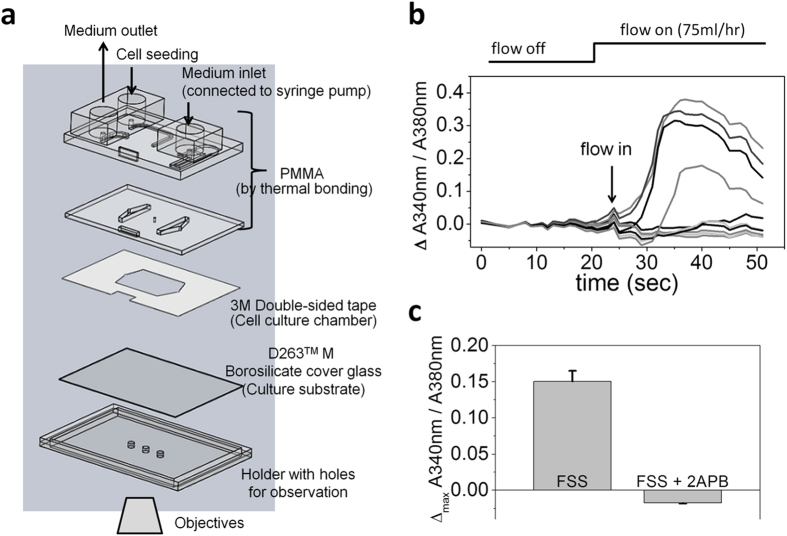
Shear flow induced the increase of intracellular free calcium in MSCs. (**a**) Components of the microfluidic device for calcium imaging. (**b**) Selected traces of changes of intracellular free calcium concentration upon shear flow activation indicated by the changes of 510 nm emission ratio of 340 nm and 380 nm excitation. Emission ratio is directly correlated to the amount of intracellular calcium. (**c**) Increase of intracellular free calcium was inhibited by 20 μM 2-APB. Value of the control set was the average of maximum changes of 510 nm emission ratio from three independent trials (mean ± SEM). Negative value of the set with 2-APB blocking was due to photo-bleaching. (Figure 5a drawn by Meng-Hua Yen).

Calcium is an important mineral to support bone remodeling. Calcium also functions as a second messenger and plays roles in cellular signaling. Since IFSS induced localization of TRPM7, which is a calcium-permeable ion channel, and promoted osteogenic-specific gene expressions, we hypothesized that the same flow rate can trigger the increase of intracellular calcium and thus induce the osteogenic differentiation of MSCs. Intracellular calcium was assessed by calcium imaging using Fura2-AM and quantified by ratiometric analysis. Ratiometric imaging depicted that fluid flow induced transient increase of calcium within 10 second after the flow started (Fig. 5b). 20 μM 2-APB was able to block the calcium flux induced by shear stress (Fig. 5c). These results exhibited the involvement of TRPM7 in the elevation of intracellular free calcium activated by shear stress.

### Blockage of TRPM7 inhibited osteogenic differentiation and proper cytoskeletal arrangement of MSCs in static culture

**Figure 6:**
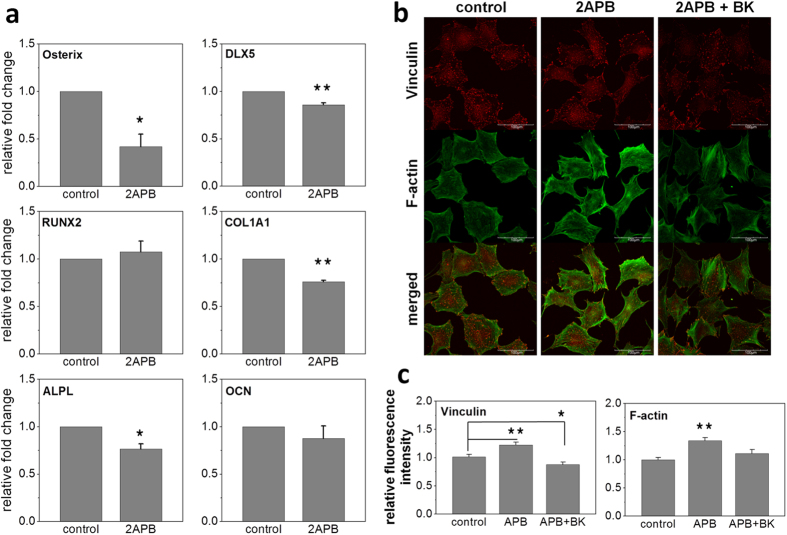
TRPM7 modulated cytoskeletal arrangement of MSCs in the early stage of osteogenic differentiation. (**a**) Relative gene expressions of MSCs on the third day of osteogenic differentiation in static condition with or without 20 μM 2-APB. Data were normalized by the respective gene expressions of MSCs without 2-APB (control). 2-APB blocking of TRPM7 downregulated gene expressions of Osterix, Dlx5, COL1A1, and ALPL. The blocking had no effect on gene expressions Runx2 and OCN. (**b**) Immunofluorescent staining of osteoblast-engaged MSCs on the third day after osteogenic induction in static condition with (middle) and without 20 μM 2-APB (left, as control) added in induction medium, as well as with 2-APB added in induction medium and cells were challenged by 10 nM bradykinin (BK) intermittently for 115 minutes (right). Scale bar = 100 μm. (**c**) Mean fluorescence intensities of vinculin and F-actin of the three groups (N = 34 for each group). Expressions of vinculin and F-actin increased and exhibited more scattered and less localized distribution in the TRPM7-inhibited group. Challenge of 10 nM Bradykinin intermittently rescued the effects caused by 2-APB. Data were normalized by control and presented as mean ± SEM. Significant difference *indicated p < 0.05 and **indicated p < 0.005.

We demonstrated that short duration of IFSS promoted osteogenic differentiation of MSCs via TRPM7 mechanosensitivity. In addition, as Chang *et al.*^26^, we also found that TRPM7 was upregulated during osteogenic differentiation of MSCs (Supplementary Figure S3). Therefore, inhibition of TRPM7 may affect osteogenic differentiation in static culture as well. Indeed, gene expressions of Osterix, Dlx5, ALPL, and COL1A1 were downregulated when the medium was supplemented with 20 μM 2-APB compared with the control group three days after the induction (Fig. 6a).

We further examined the effect of TRPM7 inhibition on cytoskeleton remodeling because it was the proposed role that TRPM7 played in cell migration^13^,^16^. After three days of osteogenic induction, immunofluorescent staining showed the increases in vinculin and F-actin expression, and the two proteins exhibited more scattered and less localized distribution in the TRPM7-inhibited group (Fig. 6a,c). After cells were intermittently incubated in 10 nM Bradykinin, a stimulator of TRPM7, to mimic IFSS (Supplementary Figure S6), the arrangement of F-actin was rescued and the cells shared similar phenotype as the control group (Fig. 6b,c). Vinculin is a cytoskeletal-associated protein participating in the formation of focal adhesion complex. It can interact with F-actin to regulate assembly and disassembly dynamics of microfilament formation and is suggested to be a mechanical mediator. Therefore, our results indicated that the regulatory mechanism of TRPM7 in the early-stage of osteogenic differentiation may be associated with actin filaments dynamics.

## Discussion

Cells react differently to different fluid flow patterns in terms of proliferation rate, morphology, or inflammatory response^33^. Despite existing discrepancies in the protocols used for studying the effects of fluid shear stress (FSS) on osteoblasts or MSCs, there is a general agreement that certain patterns of FSS promote osteogenic differentiation^34^,^35^. However, the molecular mechanisms underlying the mechanotransduction for osteogenic differentiation still remain illusive. Our results demonstrate a dynamic modulation of MSC fate commitment by a short duration of IFSS and the modulation is mediated by mechanosensitive channel TRPM7. Gene expressions of Osterix, Dlx5, ALPL and COL1A1, but not Runx2, are up-regulated after 3 hours of IFSS. Moreover, increase of Osterix gene expression is flow-rate dependent, and the upregulation diminishes when mechanical stimulus ceases or when TRPM7 is knocked-down (Figs 1c and 3b). The rapid change in gene expression of transcription factor osterix in response to mechanical stimulation highlights the intrinsic properties of mRNA instability and sensitivity in transcript levels.

The pattern of IFSS employed in the present study is designed based on the response duration of cells to chemical stimuli mediated by TRPM7^13^ and on our preliminary results of the resting time needed for reactivating calcium flux by FSS. In addition to 1.2 Pa IFSS, we have also tested the effect of 1.2 Pa continuous fluid shear stress (CFSS) on MSC osteogenic differentiation and the mechanical stimulus of CFSS up-regulates gene expression of Runx2, but not Osterix (Supplementary Figure S1). Intriguingly, recent study showed that 98 Pa pressure loading, which is another type of mechanical stimulus, induces cytosolic Ca^2+^ increase, NFATc1 (nuclear factor of activated T cells, cytoplasmic 1) nuclear localization, and the upregulation of Runx2 gene^18^. The similar gene expression patterns activated by CFSS and pressure loading indicate that the distinct mechanical stimuli may activate the same mechanotransduction pathways. However, NFATc1 is a bone-resorbing osteoclasts related gene. It controls osteoblastic bone formation only with the concomitant activation of Osterix, not Runx2^36^. Therefore, the exact mechanisms of CFSS and pressure loading to promote osteogenic differentiation of MSCs need to be further elucidated.

With characteristics similar to other channels, TRPM7 could be desensitized to prolonged constant mechanical stimulus and is either inactive or closed when the mechanical stimulation is ceased. Therefore, the mechanotransduction pathways triggered by TRPM7 activation are attenuated after MSCs stay in “stress-off” state and eventually the responding mRNA levels of transcription factors are not upregulated anymore (Fig. 1c). Repeated “stress-on” from “stress-off” state reactivates TRPM7 and therefore upregulates the downstream signals again. For example, the stressed cells collected at time point 1 (Fig. 1b), which have already experienced four of ten-minute “stress-off” states but were in a “stress-on” state, have mRNA levels of transcription factors and other osteogenic markers upregulated. The result may provide an explanation for the distinct regulatory effects on osteogenesis by CFSS and IFSS. Additionally, transient increase of intracellular free calcium under shear stress (Fig. 5b) indicates that repeated mechanical “stress-on” stimulation from “stress-off” state is important for maintaining mechanotransduction signals related to TRPM7 activities, which profiles the IFSS pattern we used in the study.

In MC3T3-E1 cells, the first response of calcium flux occurring with the onset of oscillatory flow has been suggested as extracellular calcium influx through an unidentified SACs activation^37^,^38^. Whereas the multiple intracellular calcium oscillations with lower magnitudes and higher heterogeneity occurring after the initial response are suggested to originate from intracellular stores^38^. A similar two phases of intracellular calcium increase were observed in bovine aortic endothelial cells under 65 dyn/cm^2^ continuous flow as well^39^. Recently, Ca^2+^ oscillations were also observed in human MSCs upon laser-tweezer-traction and the calcium oscillations were mediated by IP_3_-sensitive Ca^2+^channels located on the endoplasmic reticulum (ER) membrane^40^. We also observed two types of calcium fluxes activated by 12 dyn/cm^2^ shear flow in MSCs. The initial response of transient increase calcium flux showed in the present study is suggested to be TRPM7 mediated and we hypothesize that calcium oscillations occurring after the initial response are released from the ER through IP3 receptors as reported previously. The involvement of IP3 receptors in the second phase of calcium flux and their roles in osteogenesis warrants a further investigation.

C-terminal serine/threonine kinase fragments cleaved from TRPM7 are recently found to translocate to the nucleus and bind chromatin-remodeling related complexes^41^. The finding raises the possibility that coupling of ion-channel signaling and kinase activities can affect gene expression patterns. In our experiments, phosphorylated p38 and Smad1/5 showed a stronger nuclear staining after 3 hours of IFSS and the treatment of siRNA targeting TRPM7 diminished the enhancement of phosphorylation by IFSS (Fig. 4). Because TRPM7 antibody used in this study is against the amino acid residues near C-terminus of TRPM7, the nuclear staining of TRPM7 upon IFSS suggests the possibility that TRPM7 kinase fragments translocate to the nucleus and may bind to other signaling partners upon IFSS stimulation (Fig. 2 and Supplementary Figure S5c).

TRPM7 modulates focal adhesion formation and there is interplay between calcium signaling and kinase cascade triggered by TRPM7 activation in cytoskeleton remodeling^13^,^42^. Our results also indicate that TRPM7 may play a role in cytoskeleton remodeling when MSCs are undergoing osteogenic differentiation in static condition (Fig. 6b,c). We reason that TRPM7 may participate in modulating focal adhesion formation and cytoskeleton remodeling under FSS. However, even though the localization of vinculin and expression of F-actin are enhanced after 3 hours of IFSS (Fig. 2), the role of TRPM7 in mediating cytoskeleton remodeling under IFSS is still inconclusive. Because the responses of actin to FSS have been reported as cell type- and flow pattern- specific^43^,^44^,^45^, investigations in various FSS patterns and time courses are required to further confirm the relationship between TRPM7, focal adhesions and cytoskeleton.

The fact that TRPM7 gene is not upregulated by IFSS indicates that basal gene expression level of TRPM7 is sufficient for the need of protein translation under 12 dyne/cm^2^ IFSS. After being treated with siRNA, the knocked-down of TRPM7 gene expression is not enough to supply the need for TRPM7 translation induced by shear stress and the upregulation of TRPM7 gene expression was observed after siRNA treatment under shear stress (Fig. 3b). MicroRNAs, which function in RNA silencing and post-transcriptional regulation of gene expression, have been shown to play important roles in the flow-regulated mechanotransduction^46^,^47^. We are currently screening for shear-flow sensitive microRNAs and will identify the ones that interact with TRPM7 for further understanding the mechanosensitive post-transcriptional regulation.

Deletion of mouse TRPM7 gene revealed its essentiality for embryonic development^14^. Recent study of cardiac-targeted TRPM7 knockout mice also suggests that TRPM7 is critical for early embryonic cardiac development but is dispensable in late embryonic or adult myocardium^48^. In the present study, we demonstrate that TRPM7 promotes osteogenic differentiation of MSCs and the modulation is initialized by its mechanosensitivity to shear flow. Therefore, we speculate that the essentiality of TRPM7 for embryonic development maybe partially, if not all, owes to its mechanosensitivity and the role in mechanotransduction. Moreover, TRPM7 is associated with neuronal cell death under ischemic stresses and cancer invasion. Together with the recent finding of TRPM7 modulators^49^, our results may facilitate the further understanding of TRPM7-mediated cellular functions and physiological roles.

## Conclusion

We conclude that TRPM7 is mechanosensitive to intermittent shear stress (IFSS) and its activation by three hours of IFSS modulates osteogenic differentiation through TRPM7-Osterix axis. The specific mechanical cue is proposed to be transduced by calcium signaling and kinase activities. In addition, the results illustrate that the platform of microfluidic devices we used provides efficient mechano-modulation for MSC osteogenic commitment. Altogether, the application of microfluidic device with IFSS may serve as a novel platform for osteogenic induction of MSCs for skeletal tissue engineering and regenerative medicine purposes.

## Materials and Methods

### Isolation and maintenance of MSCs

MSCs were derived from the bone marrow of postnatal 7 ~ 8 week old BALB/c mouse as previously described^50^. The protocols were approved by the Taipei Veterans General Hospital Institutional Animal Care and Use Committee (IACUC). All studies involving animals were in accordance with appropriate guidelines. The isolated cells were characterized by surface markers CD11b-, CD29, CD31-, CD34, CD44, CD45-, CD73, CD105, CD106, and Sca1 using flow cytometry and assessed by the osteogenic, adipogenic, as well as chondrogenic differentiation assays before being further used for the study. For maintenance and expansion, MSCs were cultured in low glucose Dulbecco’s Modified Eagle’s Medium (Sigma-Aldrich) containing 10% fetal bovine serum (Invitrogen) and 100 U penicillin, 100 μg/mL streptomycin, 2 mM L-glutamine (1% PSG) (Sigma-Aldrich). Cells were seeded at confluency of 30% to 40% and replated using trypsin under the same culture conditions once reaching the confluency of around 70%. Cells of 12^th^- to 15^th^- passage were selectively used for the experiments.

### *In Vitro* osteogenic differentiation of MSCs

Cells were seeded at the density 1500 cells/cm^2^ with maintenance medium and changed to osteogenic induction medium on the next day. Osteogenic induction medium consists of DMEM with glucose concentration of 4500 mg/L, supplemented with 100 nM dexamethasone, 10 mM β-glycerol phosphate, 0.2 mM ascorbic acid, and 1% PSG (Sigma-Aldrich). The medium was changed twice per week. For the experiments of shear stress and calcium imaging, DMEM with 4500 mg/L glucose was replaced by DMEM with 1000 mg/L glucose.

### Quantitative real-time polymerase chain reaction

Total RNA was extracted by RNeasy Mini Kit (QIAGEN). Up to 2 μg of total RNA was reverse transcribed to complementary DNA by MMLV High Performance Reverse Transcriptase according to manufacturer’s instructions (EPICENTRE). Quantitative real-time PCR (qPCR) was performed with TaqMan® Fast Universal PCR Master Mix (2X) by Step One plus real-time PCR system (Applied Biosystems) to determine the relative gene expression profiles. The primers used for qPCR are listed in Table 1 and the gene expression of ribosomal protein S18 (RPS18) was used as an endogenous internal control.

### Microfluidic device and shear flow application

Cells were seeded on glass slides pre-coated with 0.1% type I collagen (PureCol®, Advanced BioMatrix, USA) with density of 9000 cells/cm^2^. The next day, the glass slide together with gasket insert and base plate were assembled into water-tight chambers by vacuum suction (Fig. 1a). The setup was similar to the previously described ones with some modification^33^,^51^. Inlet was connected to syringe and fluid flow was generated by programmable syringe pump (New Era Pump Systems, USA). All of the shear experiments were performed in incubators to prevent temperature perturbation. Control sets were also assembled into watertight chambers without being exposed to shear flow. Culture area of the assembled chamber was 1 cm wide (W), 4 cm long (L) and 0.09 mm thick (H). Wall shear stress τ_wall_ = 6 Qμ/WH^2^ was derived from Navier-Stokes equations and continuity equation, where Q is flow rate and μ is 0.0078 poise as the dynamic viscosity of DMEM medium^52^,^53^. Flow rates used for the study were 0.25 ml/hr, 25 ml/hr, and 75 ml/hr, which gave rise to shear stress of 0.04 dyne/cm^2^, 4 dyne/cm^2^, and 12 dyne/cm^2^ respectively, which are equal to 0.004 Pa (pascal), 0.4 Pa, and 1.2 Pa.

### Calcium imaging

High performance cover slips (Zeiss) were used for cell culturing to replace glass slides considering UV transmittance. Instead of vacuum suction for assembling, the chambers used for calcium imaging were preassembled by biocompatible 3 M double-sided tapes prior to the seeding of MSCs and one additional inlet was designed for the seeding (Fig. 5a). Cells were maintained by maintenance medium overnight with flow rate of 75 μl/hr. The next day, the temperature-controlled parallel-plate chambers were integrated with calcium imaging system (Zeiss Axio Observer with Lambda DG4). Fura-2 AM (Sigma-Aldrich) was used for ratiometric analysis (340 nm/380 nm) and data were analyzed by AxioVision 4 Physiology Module. 2-APB (Sigma-Aldrich) was added in the calcium imaging buffer (140 mM NaCl, 5 mM KCl, 2 mM CaCl2, 2 mM MgCl2, 10 mM Hepes and 15 mM Na2HPO4, pH 7.2) prior to applying shear stress without pre-incubation.

### siRNA transfection of TRPM7

siRNA with sequences targeting TRPM7 was transfected into MSCs according to the manufacturer’s instructions (Invitrogen). Briefly, 50 nM of siRNA for TRPM7 (AUAAGAUUCUGAGCCAAGUGUUUGG and CCAAACACUUGGCUCAGAAU CUUAU) or control siRNA (low GC content) was transfected with RNAiMAX transfection reagent (Invitrogen) into MSCs of 50% confluency for 24 hours at 37 °C. Cells were then washed with PBS and cultured in osteogenic induction medium for 2 hours prior to shear flow exposure.

### Immunofluorescent staining and quantification

Primary antibodies against phospho-Smad1/5 and phospho-p38 MAPK (Cell Signaling Technology, USA) were used at 1:50 dilution, TRPM7 (GeneTex, USA) and vinculin (Abcam, UK) were used at 1:100 dilution. Immunostaining was performed on static and stressed samples from a single experiment on the same day, and the images were taken by Olympus FluoView™ FV1000 confocal microscope on the same next day using the same settings of laser power and pinhole size. Quantification analysis was performed by NIH Image J using the images directly obtained from confocal microscope without any manipulation or normalization. The fluorescence intensity of each cell was obtained by mean of ROI (whole cell area) subtracted by background signal, which were from randomly-picked regions where no cell was present. Relative fluorescent intensity (signal of the stressed sample to signal of the static control from one single experiment) instead of absolute fluorescent intensity was present due to the deviation of the confocal laser power among several experimental repeats.

### Statistical analysis

All data for statistical analysis were derived from at least three independent experiments and were presented as mean ± SEM. Student’s t-test was used to compare data from two groups. *P*-value less than 0.05 were considered as statistical significant.

## Additional Information

**How to cite this article**: Liu, Y.-S. *et al.* Mechanosensitive TRPM7 mediates shear stress and modulates osteogenic differentiation of mesenchymal stromal cells through Osterix pathway. *Sci. Rep.* **5**, 16522; doi: 10.1038/srep16522 (2015).

## Supplementary Material

Supplementary Information

## Figures and Tables

**Table 1:** Sequences of RT-PCR primers for mouse MSCs.

Gene	Sequences
RPS18	CTTCCACAGGAGGCCTACAC/TGGTGTTGAGTACTCGCAAAAT
Osterix	TGCTTCCCAATCCTATTTGC/AGCTCAGGGGGAATCGAG
Runx2	GCCCAGGCGTATTTCAGA/TGCCTGGCTCTTCTTACTGAG
Dlx5	AGCCCCTACCACCAGTACG/GCTCCGCCACTTCTTTCTC
TRPM7	AGCTGCAGATTTACTAGCCTATATCC/TCTGCTGCATCAGGAAGATTT
Osteocalcin	AGACTCCGGCGCTACCTT/CTCGTCACAAGCAGGGTTAAG
COLIA1	CATGTTCAGCTTTGTGGACCT/GCAGCTGACTTCAGGGATGT
ALPL	TTAAGGGCCAGCTACACCAC/AGGGACCTGAGCGTTGGT
BMP2	CGGACTGCGGTCTCCTAA/GGGGAAGCAGCAACACTAGA
