# Supplementary material for: Mechanosensitive TRPM7 mediates shear stress and modulates osteogenic differentiation of mesenchymal stromal cells through Osterix pathway
Source: Sci Rep. 2015 Nov 12;5:16522. doi: 10.1038/srep16522 (PMC4642269; doi:10.1038/srep16522)
Supplement: Supplementary Information [file srep16522-s1.pdf]

Supplementary Information

Mechanosensitive TRPM7 mediates shear stress and modulates osteogenic differentiation of mesenchymal stromal cells through Osterix pathway

Yi-Shiuan Liu[1], Yu-An Liu[1], Chin-Jing Huang[1], Meng-Hua Yen[1], Chien-Tzu Tseng[1], Shu Chien[2,3], Oscar K. Lee [4,5,6*]

[1] Stem Cell Research Center, National Yang-Ming University, Taipei 11221, Taiwan
[2] Institute of Engineering in Medicine, University of California at San Diego, La Jolla, CA 92093, USA
[3] Departments of Bioengineering and Medicine, University of California at San Diego, La Jolla, CA 92093, USA
[4] Taipei City Hospital, Taipei 10341, Taiwan
[5] Institute of Clinical Medicine, National Yang-Ming University, Taipei 11221, Taiwan
[6] Department of Medical Research, Taipei Veterans General Hospital, Taipei 11217, Taiwan.

* Corresponding author:
Oscar Kuang-Sheng Lee, MD, PhD
Taipei City Hospital,
No. 145, ZhengZhou Road, Datong District, Taipei 10341, Taiwan
Telephone number: 886 -2 -2559 -6131
Fax number: 886 -2 -2559 -9051
E-mail address: DAV47@tpech.gov.tw

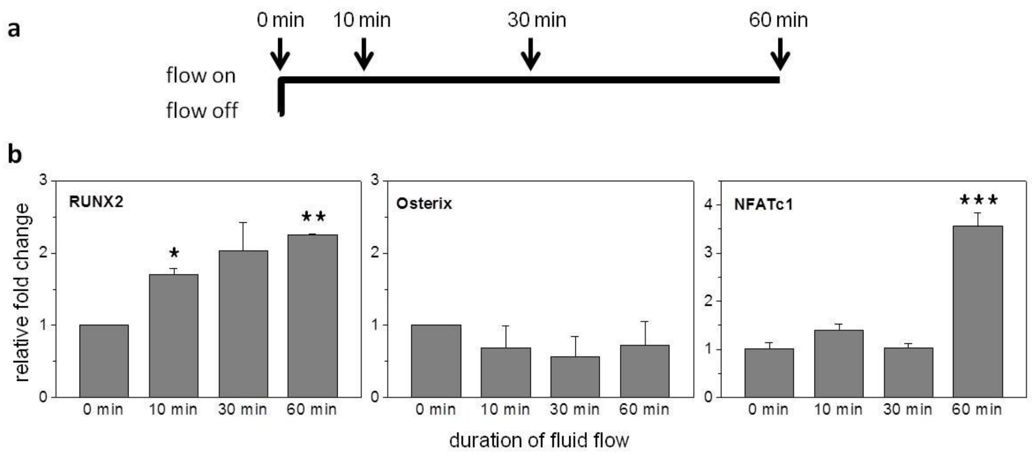

Supplementary Figure S1. Continuous shear flow upregulated Runx2 gene expression

(a) Pattern of continuous shear flow used for the study. Arrows indicate the time points at which cells were harvested when flow was on. Flow rate was 75ml/hr. (b) Relative gene expressions of Runx2, Osterix, and Nuclear factor of activated T-cells, cytoplasmic 1 (NFATc1) of MSCs under 75ml/hr continuous fluid flow for 0, 10, 30, and 60 minutes. Data were normalized by the respective gene expressions of MSCs at 0 minute (static control). Significant difference * indicates $p<0.05$ and ** indicates $p<0.005$ from data obtained in the cells of the static control.

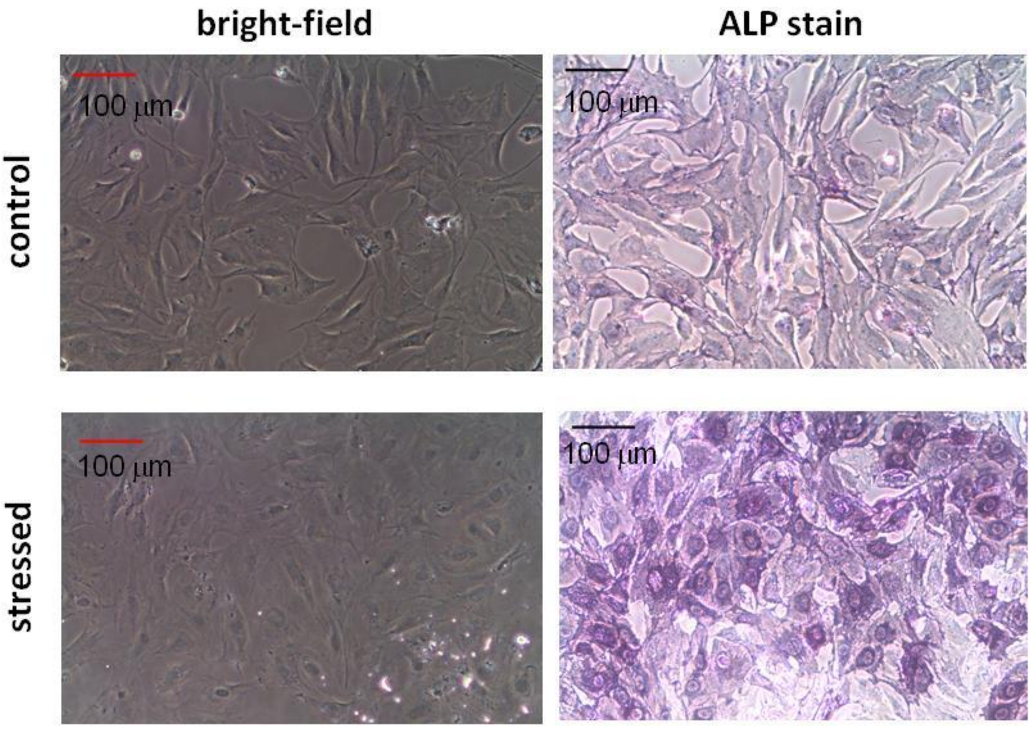

Supplementary Figure S2. Short duration of intermittent shear flow modulated osteogenic differentiation of MSCs

MSCs with and without three hours of IFSS were both maintained in induction medium afterward under 20μl/hr flow rate inside microfluidic chambers for three days. Morphology and Alkaline Phosphatase staining of MSCs after three-day induction demonstrate that MSCs with three hours of IFSS exposure (stressed) exhibited more osteoblast-like phenotypes compared to the ones without IFSS exposure (control). Scale bar = 100 μm.

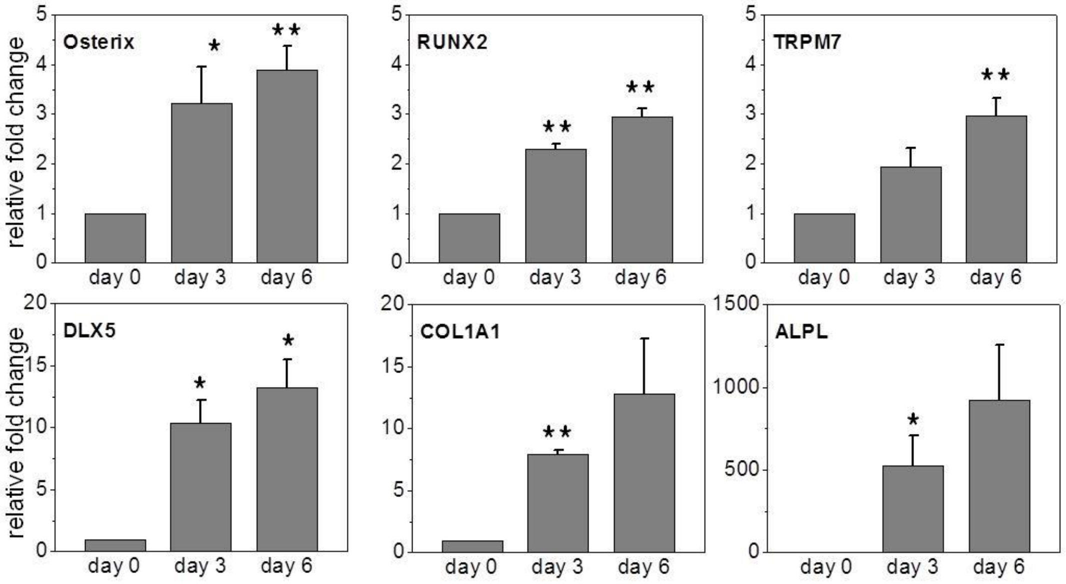

Supplementary Figure S3. Gene expression profile of mouse MSCs in the early stage of osteogenic differentiation

Gene expressions of Osterix, Runx2, Dlx5, TRPM7, COL1A1, and ALPL of undifferentiated cells (day 0), the cells under osteogenic induction for three days, as well as the cells under osteogenic induction for six days. Significant difference * indicated $p<0.05$ and ** indicated $p<0.005$ from data obtained in the undifferentiated cells.

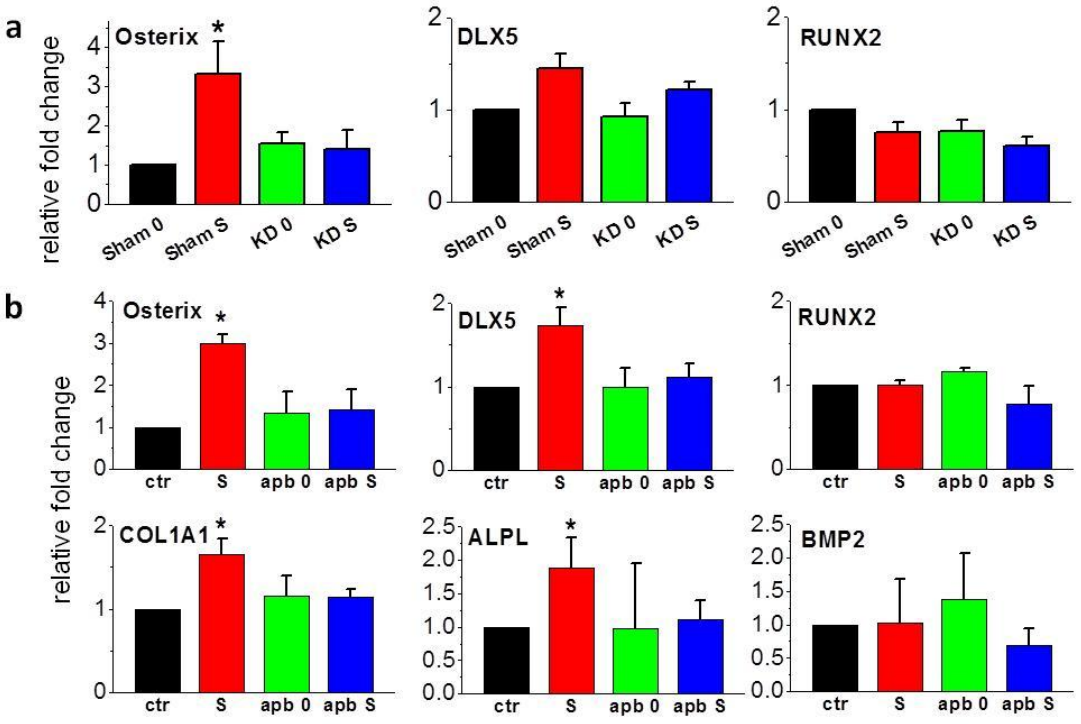

Supplementary Figure S4. TRPM7 knockdown and blocking abolished the upregulation of osteogenic marker genes under shear flow

(a) Relative gene expressions of shame control and gene knockdown MSCs with and without intermittent shear stress (IFSS). Sham 0 and Sham S: scrambled siRNA control without and with IFSS. KD 0 and KD S: TRPM7 knockdown without and with IFSS. (b) Relative gene expressions with (apb 0 and apb S) and without (ctr and S) 100 μM 2-APB additive. Data were normalized by the respective gene expressions of control group (Shame 0 in (a) and ctr in (b)). Significant difference * indicated $p<0.05$.

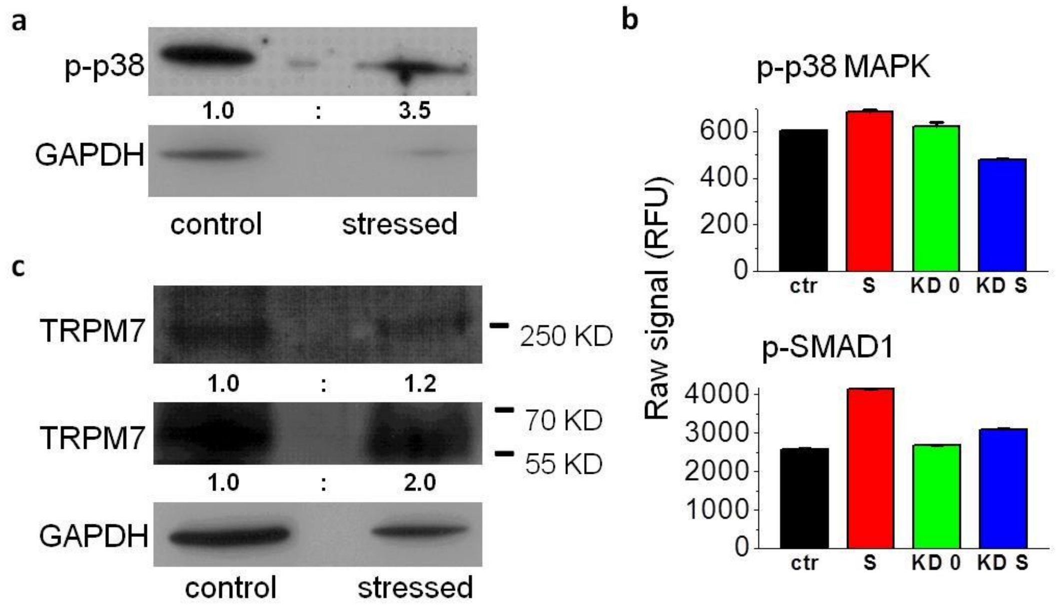

Supplementary Figure S5. Shear stress induced phosphorylation of Smad1/5 and p38 MAPK and upregulated TRPM7 protein expression

(a) Western blotting of p-p38 MAPK. Numbers indicate the ratio of control to stressed p-p38 signals normalized by GAPDH. Signals were semi-quantified by Image J. (b) Levels of p-p38 and p-SMAD1 were detected using PhosphoTracer ELISA Kits (abcam). Each of raw signals was from 4.5μg of total protein pooled from at least three microfluidic chips. ctr: static control. S: IFSS. KD 0: TRPM7 knockdown static. KD S: TRPM7 knockdown IFSS. (c) Western blotting of TRPM7 with (stressed) and without (control) IFSS. Numbers indicate the ratio of control to stressed TRPM7 (full-length and cleaved C-term) signals normalized by GAPDH.

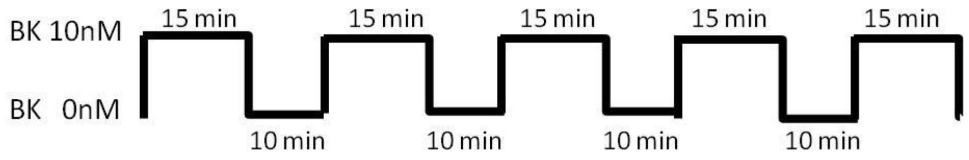

Supplementary Figure S6. Pattern used for Bradykinin application

10 nM Bradykinin was intermittently added into the induction medium on the third day of osteogenic differentiation.
